# Paraspinal muscle parameters’ predictive value for new vertebral compression fractures post-vertebral augmentation: Nomogram development and validation

**DOI:** 10.3389/fmed.2024.1379078

**Published:** 2024-05-15

**Authors:** Ming Tang, Guangdong Zhang, Fanyi Zeng, Xindong Chang, Qingqing Fang, Mingfei He, Shiwu Yin

**Affiliations:** ^1^Department of Interventional Vascular Medicine, The Second People’s Hospital of Hefei, Hefei Hospital Affiliated to Anhui Medical University, Hefei, Anhui, China; ^2^The Fifth Clinical College of Medicine, Anhui Medical University, Hefei, Anhui, China

**Keywords:** osteoporotic vertebral compression fractures, percutaneous vertebroplasty, percutaneous kyphoplasty, new vertebral compression fractures, paraspinal muscle

## Abstract

**Objective:**

Prior research underscores the significance of paraspinal muscles in maintaining spinal stability. This study aims to investigate the predictive value of paraspinal muscle parameters for the occurrence of new vertebral compression fractures (NVCF) following percutaneous vertebroplasty (PVP) or percutaneous kyphoplasty (PKP) in patients with osteoporotic vertebral compression fractures (OVCF).

**Methods:**

Retrospectively collected data from October 2019 to February 2021 (internal validation, *n* = 235) and March 2021 to November 2021 (external validation, *n* = 105) for patients with OVCF treated with PVP/PKP at our institution. They were randomly divided into training (188 cases) and validation groups (47 cases) at an 8:2 ratio. Lasso regression and multivariable logistic regression identified independent risk factors in the training set, and a Nomogram model was developed. Accuracy was assessed using receiver operating characteristic curves (ROC), calibration was evaluated with calibration curves and the Hosmer-Lemeshow test, and clinical utility was analyzed using decision curve analysis (DCA) and clinical impact curve (CIC).

**Results:**

Surgical approach, spinal computed tomography (CT) values, and multifidus skeletal muscle index (SMI) are independent predictors of postoperative NVCF in OVCF patients. A Nomogram model, based on the identified predictors, was developed and uploaded online. Internal validation results showed area under the curve (AUC) values of 0.801, 0.664, and 0.832 for the training set, validation set, and external validation, respectively. Hosmer-Lemeshow goodness-of-fit tests (*χ*^2^ = 7.311–14.474, *p* = 0.070–0.504) and calibration curves indicated good consistency between observed and predicted values. DCA and CIC demonstrated clinical net benefit within risk thresholds of 0.06–0.84, 0.12–0.23, and 0.01–0.27. At specificity 1.00–0.80, the partial AUC (0.106) exceeded that at sensitivity 1.00–0.80 (0.062).

**Conclusion:**

Compared to the spinal CT value, the multifidus SMI has certain potential in predicting the occurrence of NVCF. Additionally, the Nomogram model of this study has a greater negative predictive value.

## 1 Introduction

Osteoporosis, defined by the World Health Organization as a bone mineral density (BMD) T-score < −2.5 measured through dual-emission x-ray absorptiometry (DXA), is a prevalent condition affecting 30% of women and 12% of men (1). Osteoporosis signifies compromised bone mass and reduced strength, thereby elevating the risk of complications such as osteoporotic vertebral compression fractures (OVCF) and spinal deformities. OVCF stands as the most common osteoporotic fracture globally, with an incidence of approximately 30–50% in individuals aged 50 and above (2). Primary treatments for OVCF include percutaneous vertebroplasty (PVP) and percutaneous kyphoplasty (PKP). There is ongoing debate about which technique is superior. Some studies indicate that PKP can significantly increase the vertebral height after surgery and reduce the risk of cement leakage (3). Despite the maturity of these minimally invasive techniques, postoperative complications such as new vertebral compression fractures (NVCF) warrant attention, with reported incidence ranging from 2 to 52% (4, 5). Existing studies indicate age, gender, body mass index (BMI), BMD, and cement leakage as potential risk factors for NVCF (6, 7).

Given the spine’s role as a complex, multi-joint structure, its stability is crucial for maintaining normal posture control and trunk movement. Panjabi (8) defined that spinal stability is determined by the complex interaction of three systems: the passive subsystem (vertebrae, intervertebral discs, facet joints, spinal ligaments), the active subsystem (paraspinal muscles), and the neural control subsystem. Dysfunction in any component of this system can alter stability, leading to pathological decompensation. Osteoporosis and sarcopenia, prevalent musculoskeletal disorders in the aging population, often coexist. With the global aging trend, their incidence is expected to rise (9). In sarcopenia, degeneration of paraspinal muscles (fat infiltration, fibrosis, and atrophy) reduces spinal stability, increasing the risk of OVCF (10). Concurrently, existing research confirms magnetic resonance imaging (MRI) as a gold standard for assessing overall and local skeletal muscle mass, subcutaneous adipose tissue, and visceral adipose tissue. In OVCF patients, MRI not only reveals vertebral morphological changes but also clearly displays post-fracture traumatic bone marrow edema (11). This offers an opportunity for assessing skeletal muscle mass in such patients. Several studies have identified a correlation between MRI-measured paraspinal muscle fat infiltration and NVCF (11–13). Infiltration of muscle fat leads to functional muscle reduction, inevitably affecting spinal stability. Therefore, this study aims to investigate the predictive value of MRI-assessed paraspinal muscle parameters for NVCF following PVP/PKP and to develop and validate a corresponding Nomogram. This study strictly adheres to the guidelines of the “Transparent Reporting of a Multivariable Prediction Model for Individual Prognosis or Diagnosis (TRIPOD),” with Supplementary materials providing detailed information.

## 2 Methods

### 2.1 Patients

This retrospective study selected OVCF patients treated with PVP/PKP in our hospital from October 2019 to November 2021. The internal validation set comprises cases from October 2019 to February 2021, and the external validation set includes cases from March 2021 to November 2021. Post PVP/PKP, NVCF was diagnosed based on recurring back pain, especially during movement, and confirmed by new wedge changes on X-rays or MRI (Supplementary Figure S1). Inclusion criteria: (1) Age ≥ 60 years; (2) Preoperative diagnostic support for OVCF through X-ray, three-dimensional reconstruction of spinal computed tomography (CT), and MRI; (3) Underwent vertebroplasty for the first time; (4) DXA-measured BMD *T*-score < −2.5. Exclusion criteria: (1) symptomatic pain due to other causes such as disc herniation or spondylolisthesis; (2) Fractures from high-energy trauma or tumor-related fractures; (3) History of previous lumbar spine surgery or clear trauma; (4) Neuro-musculoskeletal or endocrine disorders affecting paraspinal muscle function, or long-term use of steroid medications causing skeletal metabolism abnormalities; (5) Incomplete clinical data; and (6) Coexisting infections, severe cardiovascular or cerebrovascular diseases, or other congenital conditions.

### 2.2 Imaging assessment method

#### 2.2.1 Paraspinal muscle

**Figure 1:**
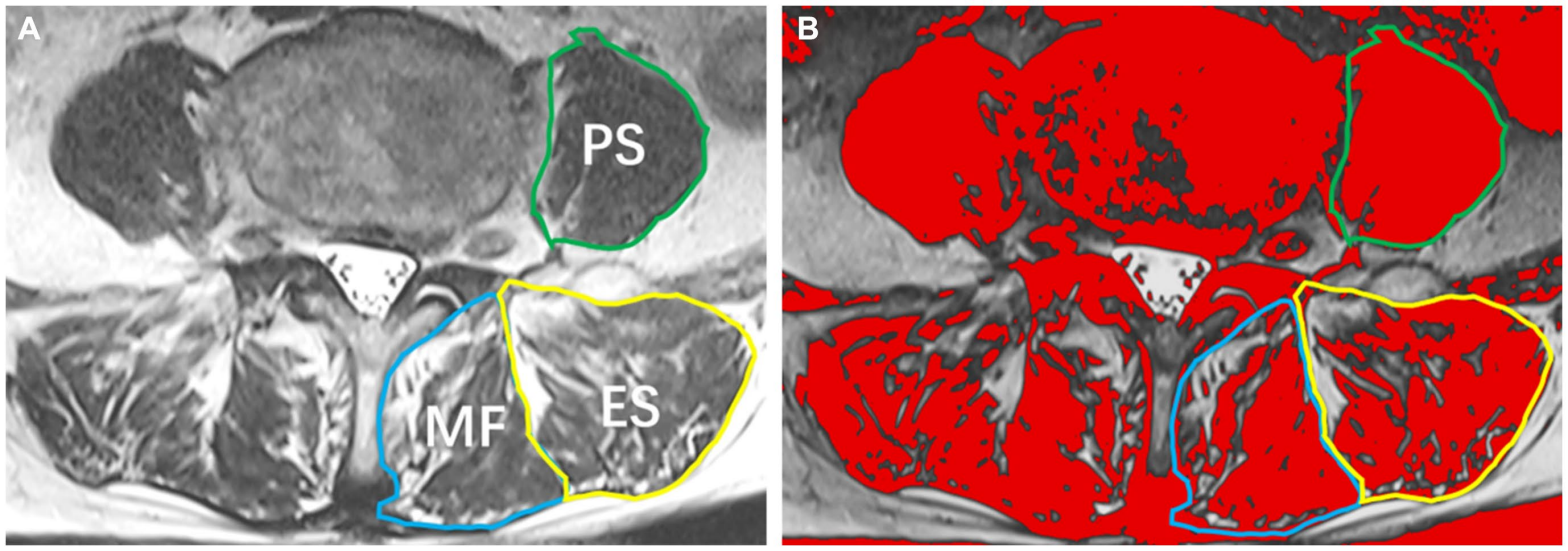
Paraspinal muscles at the L4/5 Level. **(A)** T2-weighted image illustrating the multifidus muscle (MF), erector spinae muscle (ES), and psoas muscle (PS); **(B)** Image J analysis depicting the cross-sectional area of paraspinal muscles and the extent of fatty infiltration.

Participants underwent preoperative examination using a 3.0 T MRI system, obtaining supine T2-weighted axial images. The maximum cross-sectional area (CSA) of the paraspinal muscles is typically located between the L3/L4 and L4/L5 disc levels based on anatomical features, while the largest CSA of the psoas major muscle is identified at the L4/L5 disc level (14). Therefore, muscle measurements in this study focused on the paraspinal muscles at the L4/5 disc level. Using the sample function in R Studio, 30 patients were randomly selected without replacement. Their T2-weighted axial images were imported into Image J software for measurement by two radiologists blinded to the patients’ outcomes (Reader 1, with 15 years of experience in MRI diagnosis; Reader 2, with 8 years of experience). The preoperative CSA of the bilateral multifidus/erector spinae/psoas major muscles and the fat CSA of these muscles were measured (Figure 1). After 1 month, Reader 2 performed repeated measurements for the same 30 patients. In cases where intra-and inter-observer correlation coefficients were ≥ 0.75, it indicated good consistency in the measurements between the two readers and for the same reader. Subsequently, all paraspinal muscle data were independently measured by Reader 2. Functional cross-sectional area (FCSA) was obtained by calculating the difference between paraspinal muscle CSA and paraspinal muscle fat CSA. To standardize for variations in patient height, the skeletal muscle index (SMI) for paraspinal muscles was calculated as Paraspinal Muscle SMI = Paraspinal Muscle FCSA (mm^2^) ÷ Height2 (m^2^). The average SMI of paraspinal muscles on both sides was used as the paraspinal muscle SMI.

#### 2.2.2 Fractured vertebral characteristics

Reader 2 measured, from lateral X-ray images, OVCF segment (thoracic segment < T11, thoracolumbar segment T11-L2, lumbar segment > L2), fracture shape (wedge, biconcave, crush), fracture compression percentage, pre−/postoperative anterior vertebral height (AVH), preoperative adjacent upper and lower vertebral average AVH of the compressed vertebra, pre−/postoperative Cobb angles (formed by the upper and lower endplates of the compressed vertebra), and cement leakage. Using these measurements, additional calculations were performed, including the anterior vertebral height ratio (AVHR) = Compressed vertebra AVH ÷ Average AVH of adjacent upper and lower vertebrae × 100%, anterior vertebral height recovery ratio (AVHRR) = Postoperative AVHR − Preoperative AVHR, and Cobb angle change = Preoperative Cobb angle − Postoperative Cobb angle. Postoperative lateral X-ray images of all patients were obtained within 3 days after the surgery.

Using MRI and three-dimensional reconstruction of spinal CT, we measured intravertebral clefts (IVC) and spinal CT values. IVC were identified in CT as gas density within the vertebral body, displaying characteristic “double-line sign” on MRI with T1-weighted low signal and T2-weighted high signal. Spinal CT values were determined in the adjacent vertebral body of the fractured vertebra. The vertebral body was evenly divided into three sections in the sagittal plane, and the average CT value was calculated in the maximum elliptical region of interest containing only trabecular bone (Supplementary Figure S1).

### 2.3 Imaging assessment method

Incorporated risk factors encompassed general characteristics such as age, gender, education level, occupation, weight, BMI, diabetes, hypertension, smoking, drinking, preoperative Visual Analog Scale (VAS) score, postoperative VAS score, and time to first ambulation post-surgery. Treatment measures included anti-osteoporosis treatment, surgical approach, puncture pathway, Volume of injected bone cement, and duration of surgery. Vertebral features comprised multiple vertebral fractures, fracture segment, fracture compression percentage, fracture shape, IVC, spinal CT values, AVHRR, Cobb angle changes, and cement leakage. Laboratory examinations involved white leukocyte, hemoglobin, urea, creatinine, C-reactive protein/albumin ratio (CAR). Parameters of paraspinal muscles included multifidus/erector spinae/psoas major SMIs. A 2-year follow-up utilized outpatient visits, electronic medical record tracking, and telephone follow-ups. Anti-osteoporotic treatment referred to regular use of bisphosphonates during the follow-up period.

### 2.4 Statistical methods

All statistical analyses were conducted using R studio software (v4.2.3, http://www.rproject.org/). Bonferroni correction was applied using the “agricolae” package. Intra-and inter-observer correlation coefficients (ICC) was assessed using the “irr” package, with results ≥0.75 considered indicative of good consistency. Predictive factors for NVCF occurrence were selected through Least Absolute Shrinkage and Selection Operator (LASSO) regression using the “glmnet” package. Logistic regression was performed using the “rms” package. The Nomogram was plotted using the “nomogramFormula” package. ROC curves (receiver operating characteristic curve) and partial ROC (pROC) were generated using the “pROC” package. The “epiR” package was utilized for confusion matrix analysis to evaluate model predictive performance. Calibration curves and the Hosmer-Lemeshow goodness-of-fit test were produced using the “rms” package. Decision Curve Analysis (DCA) and Clinical Impact Curve (CIC) were plotted using the “rmda” package. Statistical significance was set at *p* < 0.05. Additional details are available in Supplementary information S3.

## 3 Results

### 3.1 General results

The internal validation set included 235 patients, comprising 53 males and 182 females, with an age range of 62–97 years and a mean age of 75.04 ± 7.97 years (The comparison between NVCF and No-NVCF patients is shown in Supplementary Table S1, where there is no significant difference in the occurrence of NVCF between patients in the PVP group and those in the PKP group, *p* = 0.065). Among these patients, 37 experienced NVCF, resulting in a recurrence rate of 15.74%. The external validation set comprised 105 patients, with 30 males and 75 females, aged 60–93 years, and a mean age of 75.60 ± 8.59 years. NVCF occurred in 9 cases, yielding a recurrence rate of 8.57%. The intra-and inter-observer correlation coefficients were between 0.829 and 0.989, indicating good consistency. Utilizing the “createDataPartition” function in the “caret” package of the R, the internal validation set was randomly allocated into training (*n* = 188) and validation (*n* = 47) sets at an 8:2 ratio (Table 1). The detailed process of internal validation dataset partitioning is presented in Supplementary information S4, Supplementary Table S4, and Supplementary Figure S2.

**Table 1:** Comparison of characteristics among the training, testing, and validation sets.

Characteristic	Training set (*n* = 188)	Testing set (*n* = 47)	Validation set (*n* = 105)	*p*
Age (year)	75.01 ± 8.22	75.17 ± 6.95	75.60 ± 8.59	0.559
Gender (male)	44 (22.79%)	9 (30.88%)	30 (%)	0.304
Body weight (kg)	56.68 ± 10.22	55.28 ± 9.20	56.72 ± 10.88	0.312
BMI (kg/m^2^)	22.18 ± 3.75	21.88 ± 2.69	21.79 ± 3.36	0.227
Diabetes (yes)	27 (14.71%)	10 (19.12%)	16 (%)	0.347
Hypertension (yes)	86 (47.79%)	26 (44.12%)	48 (%)	0.311
Smoking (yes)	11 (5.15%)	1 (2.94%)	4 (%)	0.505
Drinking (yes)	18 (9.93%)	6 (14.71%)	13 (%)	0.582
Pre-VAS score*	3 (3, 5)	3 (3, 4.5)	3 (3, 4)	0.081
Post-VAS score*	2 (1, 3)	2 (2, 3)	2 (1, 3)	0.579
Educational level				0.271
Primary school≤	151 (77.21%)	34 (73.53%)	75 (%)	
Secondary school	2 (1.10%)	0 (0.00%)	1 (%)	
High school	26 (16.54%)	9 (20.59%)	24 (%)	
≥College	9 (5.15%)	4 (5.88%)	5 (%)	
Occupation				0.594
Farmer	71 (37.13%)	14 (30.88%)	37 (%)	
Laborer	5 (2.21%)	1 (4.41%)	2 (%)	
Self-employed households	5 (2.57%)	0 (1.47%)	5 (%)	
Retirement	75 (40.07%)	23 (52.94%)	47 (%)	
Other	32 (18.01%)	9 (10.29%)	15 (%)	
Time to first ambulation (day)	2 (1, 3)	2 (1, 3)	2 (1, 3)	0.137
Osteoporosis medication	33 (15.07%)	7 (14.71%)	11 (%)	0.146
Surgical approach				0.682
PVP	68 (63.24%)	18 (69.12%)	35 (%)	
PKP	120 (36.76%)	29 (30.88%)	70 (%)	
Puncture pathway				0.115
Single	34 (19.49%)	13 (23.53%)	21 (%)	
Both	154 (80.51%)	33 (76.47%)	84 (%)	
Volume of injected bone cement (ml)*	5 (3.69, 5)	5 (4, 6)	4 (3, 5)	0.196
Duration of surgery (min)	43.61 ± 16.17	44.66 ± 15.45	41.15 ± 14.69	0.129
Multiple vertebral fractures	28 (14.71%)	11 (17.65%)	13 (%)	0.138
Fracture segment				**<0.001**^#^
T10≤	27 (9.93%)	5 (7.35%)	0 (%)	
T11–L2	139 (75.00%)	35 (70.59%)	78 (%)	
L3–L5	22 (15.07%)	7 (22.06%)	27 (%)	
Fracture compression (%)	40.63 ± 11.72	41.21 ± 11.20	38.90 ± 12.38	0.236
Fracture shape				**0.009** ^†^
Wedge	114 (54.78%)	29 (55.88%)	44 (%)	
Biconcave	71 (43.75%)	18 (42.65%)	59 (%)	
Crush	3 (1.47%)	0 (1.47%)	2 (%)	
IVC	46 (25.37%)	14 (19.12%)	22 (%)	0.328
Spinal CT values (HU)	59.33 ± 29.75	56.00 ± 37.73	56.23 ± 31.71	0.173
AVHRR (%)*	11.57 (3.79, 18.60)	8.52 (6.14, 18.67)	8.62 (3.97, 16.01)	0.171
Cobb angle change (°)*	2.18 (0.49, 4.22)	2.51 (1.21, 4.49)	2.30 (0.62, 4.52)	0.180
Cement leakage (yes)	75 (37.50%)	22 (51.47%)	40 (%)	0.406
Leukocyte (10^9^/L)	6.22 ± 1.80	6.29 ± 2.40	6.39 ± 2.33	0.577
Hemoglobin (g/L)	120.84 ± 14.97	120.28 ± 15.00	120.01 ± 14.72	0.719
Urea (mmol/L)*	6.05 (4.83, 7.52)	6.13 (5.48, 7.30)	6.18 (5.03, 7.59)	0.197
Creatinine (μmol/L)	61.95 ± 20.35	62.84 ± 17.08	65.04 ± 22.17	0.199
CAR*	0.16 (0.08, 0.49)	0.15 (0.08, 0.73)	0.16 (0.06, 0.57)	0.890
SMI (mm^2^/m^2^)				
Multifidus	142.93 ± 62.53	147.91 ± 51.79	145.14 ± 58.49	0.658
Erector spinae	319.33 ± 121.26	353.48 ± 122.87	337.65 ± 118.59	0.134
Psoas major	291.44 ± 88.02	287.19 ± 106.62	290.78 ± 90.04	0.381

Bold typeface indicates statistical significance. BMI, body mass index; VAS, visual analog scale; PVP, percutaneous vertebroplasty; PKP, percutaneous kyphoplasty; IVC, intravertebral cleft; AVHRR, anterior vertebral height recovery ratio; CAR, C-reactive protein/albumin ratio; SMI, skeletal muscle index.

*Median (P25, P75); ^#^Statistical differences exist between the validation set and the training/testing sets; ^†^Statistical differences exist between the training set and the validation set.

### 3.2 General result the difference of paraspinal muscle SMI in gender and NVCF groups and its correlation with other predictive factors

Grouped by the occurrence of NVCF, the paraspinal muscle SMI in the internal validation set showed no statistical differences (*p* = 0.212–0.714), as detailed in Supplementary Table S1. Upon grouping the internal validation set by gender, the multifidus SMI was 145.42 ± 60.76 for males and 143.49 ± 60.54 for females (*p* = 0.937). The erector spinae SMI was 302.10 ± 111.08 for males and 333.16 ± 124.53 for females (*p* = 0.085). Additionally, the lumbar erector spinae SMI was 285.30 ± 84.75 for males and 292.14 ± 93.93 for females (*p* = 0.765).

In the correlation analysis, the multifidus SMI was positively correlated with hemoglobin (R = 0.150) in all patients (235) of the internal validation set, the erector spinae SMI was positively correlated with hemoglobin (R = 0.212), urea (R = 0.132) and creatinine (R = 0.162), and the psoas major SMI was positively correlated with hemoglobin (R = 0.269) and creatinine (R = 0.247). In the NVCF patients (37) of the internal validation set, the multifidus SMI was negatively correlated with vertebral compression percentage (R = -0.331), the erector spinae SMI was positively correlated with hemoglobin (R = 0.466), and the psoas major SMI was positively correlated with creatinine (R = 0.362) and spinal CT value (R = 0.336) (Supplementary Table S3).

### 3.3 Nomogram model development

**Figure 2:**
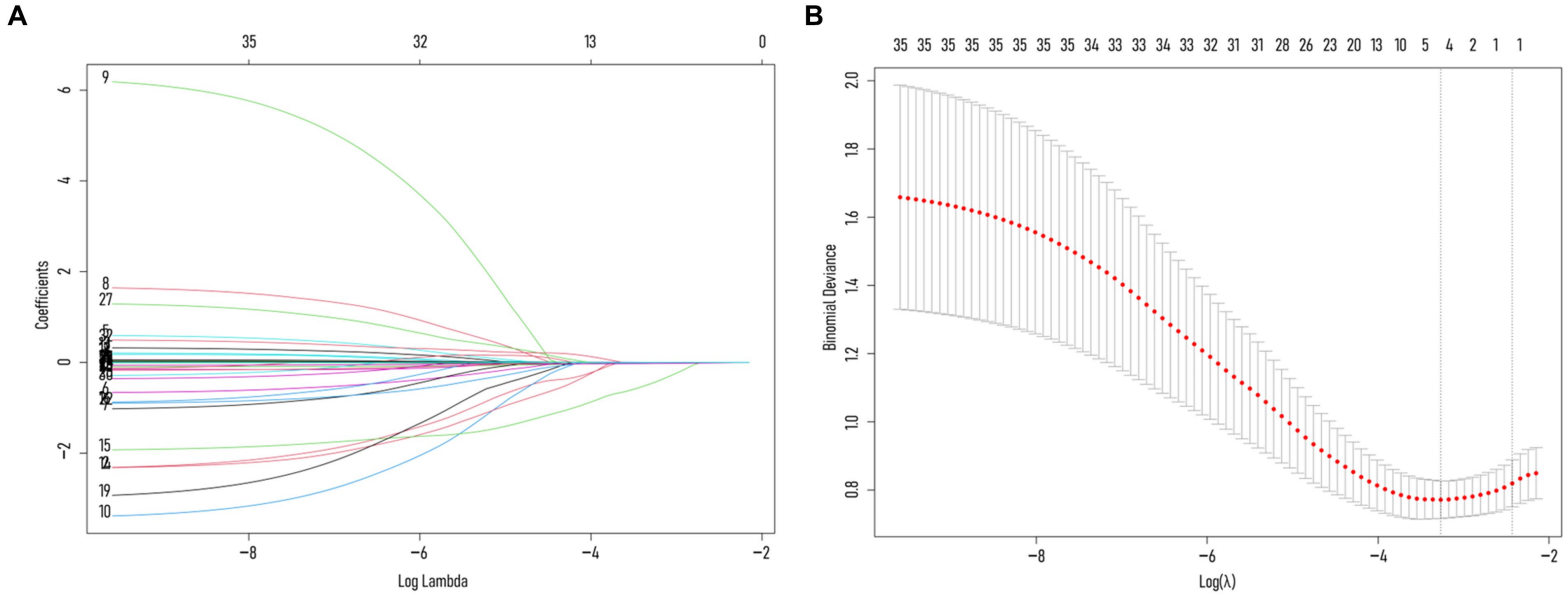
**(A)** Characterizing the variations of LASSO regression coefficients. **(B)** LASSO regression selects the optimal parameter lambda through cross-validation. The dashed line on the right represents lambda values with average error within ±1 standard deviation, indicating improved model performance.

**Figure 3:**
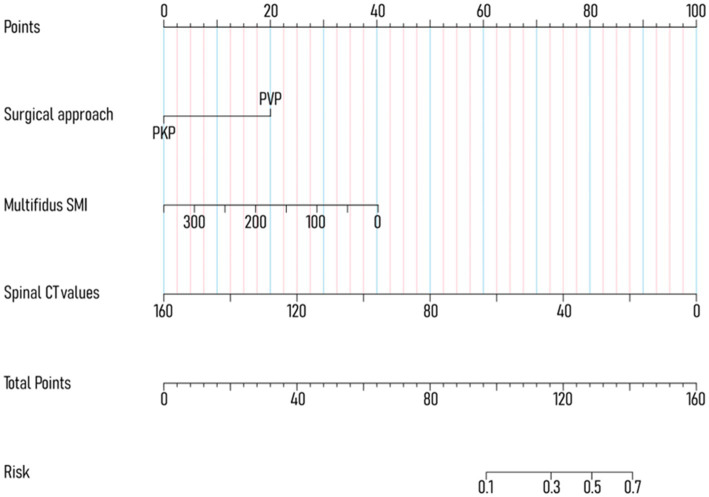
Developed Nomogram based on multifactorial logistic regression analysis.

To avoid overfitting, each feature required at least 10–15 patients for model development (15, 16). With 188 patients in the training set, the maximum feature limit was set at 18. Lasso regression was employed for variable selection. The model performed well with five predictors at lambda = 0.038 (Figure 2). Body weight, surgical approach, duration of surgery, spinal CT values, and multifidus SMI were included in the logistic regression model, revealing that surgical approach, spinal CT values, and multifidus SMI (*p* < 0.05) were independent predictors for post-vertebroplasty NVCF (Table 2). A Nomogram model was developed using these three predictors (Figure 3) and made available at https://sofarnomogram.shinyapps.io/NVCFnomogram/.

**Table 2:** Logistic regression analysis of risk factors for NVCF occurrence in the training set patients.

Variable	B	S.E	*p*	OR	OR 95%CI	
					Lower	Upper
Surgical approach						
PVP	REF					
PKP	1.390	0.485	0.004	0.249	0.092	0.630
Spinal CT value	−0.043	0.010	<0.001	0.958	0.937	0.976
Multifidus SMI	−0.008	0.004	0.045	0.992	0.984	0.999

REF, reference variable; PVP, percutaneous vertebroplasty; PKP, percutaneous kyphoplasty; SMI, skeletal muscle index.

### 3.4 Nomogram model validation

**Figure 4:**
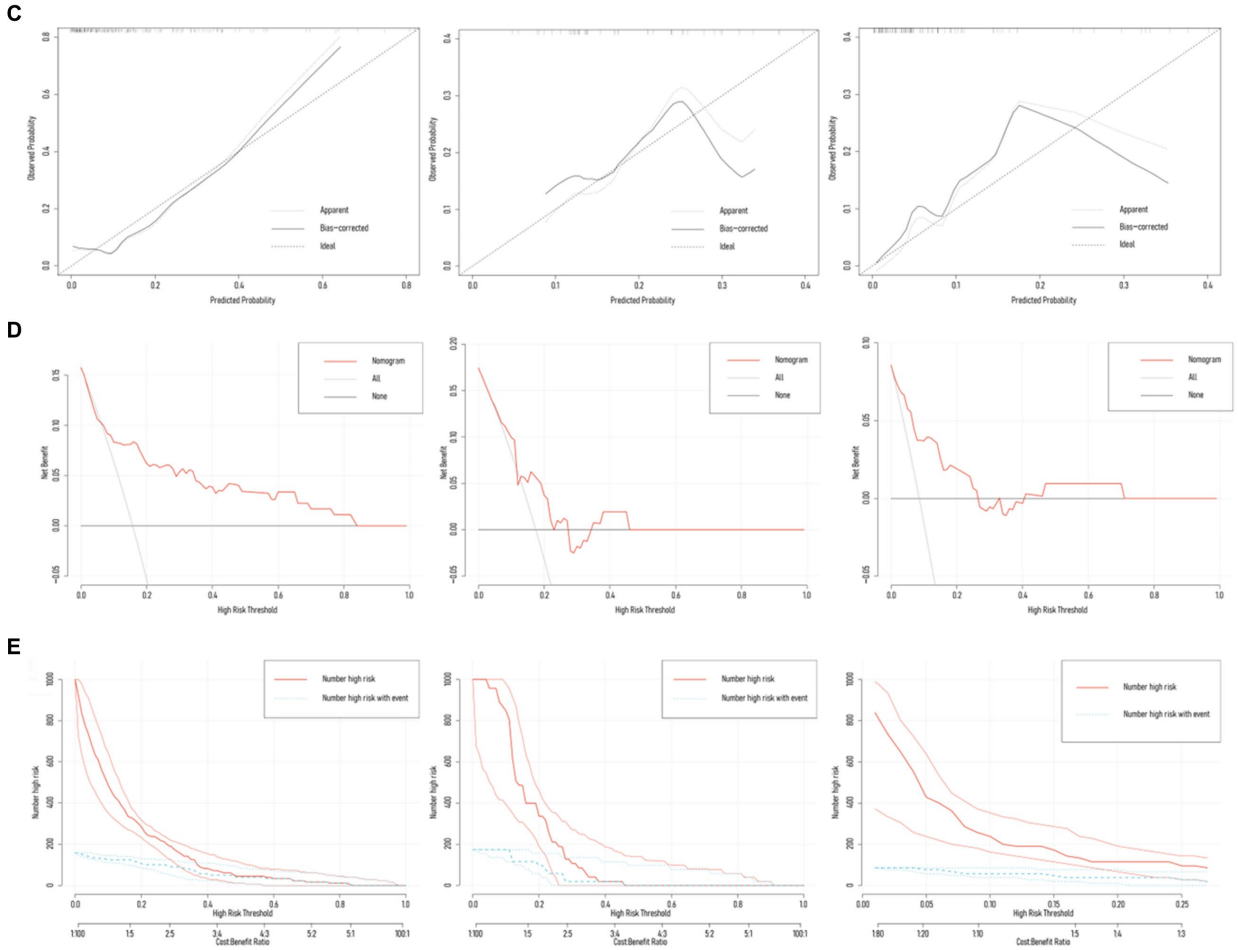
Diagnostic performance of the Nomogram model. **(A)** Nomogram model predicted the probability of NVCF in the training set (*n* = 188), testing set (*n* = 47), and validation set (*n* = 105), compared with actual diagnoses. The dot-dash line represents the cutoff value. **(B)** AUC of the ROC curves for the three sets, with Delong test *p*-values ranging from 0.134 to 0.688. **(C)** Calibration curves for the three sets, with Hosmer-Lemeshow goodness-of-fit test *p*-values ranging from 0.070 to 0.504. **(D,E)** Decision curve analysis (DCA) and clinical impact curve (CIC) demonstrate the net benefit in the low-risk population for the training, testing, and validation sets.

The training set exhibited an AUC of 0.801 (95% *CI* 0.698–0.905); the testing set, 0.664 (95% *CI* 0.475–0.853); and validation set, 0.832 (95% *CI* 0.724–0.941). Delong test revealed *p*-values ranging from 0.134 to 0.688 among the three (Table 3; Figure 4; Supplementary Figure S3). The training set’s Hosmer-Lemeshow goodness-of-fit test was *χ^2^* = 14.474, *p* = 0.070; the testing set’s was *χ^2^* = 9.216, *p* = 0.324; and the validation set’s was *χ^2^* = 7.311, *p* = 0.504. Calibration curves for the three groups are depicted in Figure 4.

**Table 3:** Diagnostic performance of Nomogram model in the training, testing, and validation sets.

	AUC (95%CI)	Accuracy	Sensitivity (95%CI)	Specificity (95%CI)	PPV (95%CI)	NPV (95%CI)
Training set	0.801 (0.698, 0.905)	0.761	0.786 (0.590, 0.917)	0.756 (0.682, 0.821)	0.361 (0.242, 0.494)	0.953 (0.900, 0.982)
Testing set	0.664 (0.475, 0.853)	0.702	0.667 (0.299, 0.925)	0.711 (0.541, 0.846)	0.353 (0.142, 0.617)	0. 900 (0.735, 0.979)
Validation set	0.832 (0.724, 0.941)	0.610	0.889 (0.518, 0.997)	0.583 (0.478, 0.683)	0.167 (0.075, 0.302)	0.982 (0.906, 1.000)

AUC, area under the receiver operating characteristic curve; PPV, positive predictive value; NPV, negative predictive value.

### 3.5 Nomogram model’s clinical utility

**Figure 5:**
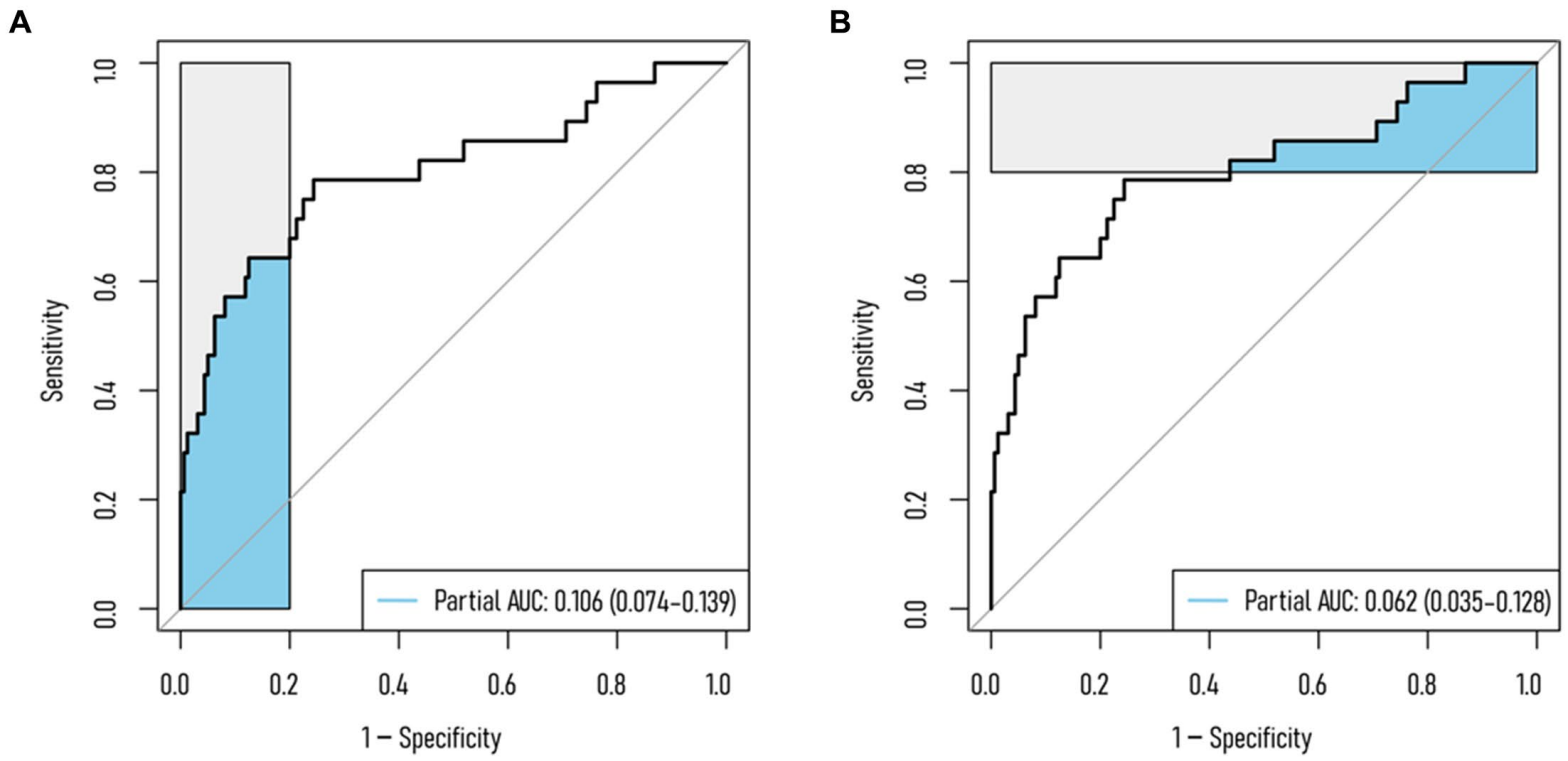
Partial AUC (pAUC) for the training set. **(A)** pAUC at 1–0.80 specificity; **(B)** pAUC at 1–0.80 sensitivity.

Results from DCA curves in the training, testing, and validation sets indicated maximal clinical net benefit at risk thresholds of 0.06–0.84, 0.12–0.23, and 0.01–0.27, respectively. The predictive model demonstrated significant additional clinical net benefit in identifying low-risk cases of NVCF within these ranges (Figure 4). Risk stratification for 1,000 predictions using CIC revealed consistently higher predicted cases of NVCF compared to actual occurrences within the threshold probability range (Figure 4). The pAUC_SP_ (0.106) at specificity 1.00–0.80 surpassed pAUC_SE_ (0.062) at sensitivity 1.00–0.80, with a Bootstrap *p*-value (5,000 repetitions) of 0.044 (Figure 5).

## 4 Discussion

In our study, we found that when the training set and testing set were divided in an 8:2 ratio, multifidus SMI became a protective factor for NVCF occurrence. Compared with 6:4 (spinal CT value + surgical approach), Delong test *p*-value was 0.215, and there was no significant difference in pAUC between the two models at specificity and sensitivity of 1–0.75 and 1–0.80 (*p* = 0.644–0.783). To explore the impact of paraspinal muscle SMI on NVCF occurrence, we chose the model with 8:2 ratio division. Previous studies have shown that gender, BMI, duration of surgery, bone cement leakage and IVC are predictive factors for NVCF, so we performed subgroup analysis, and the results showed that the model was not affected by these factors (AUC: 0.703–0.837; *p* = 0.232–0.977). At the same time, we also found that postoperative time to first ambulation (≤3 days) did not affect the model.

Osteoporosis, characterized by bone loss and disrupted microstructure, renders bones fragile and prone to low-energy trauma. The spine and hips are common sites for osteoporotic fractures, affecting about half of postmenopausal women (17). Notably, vertebral fractures escalate the risk of NVCF by 4–7 times, with increasing risk correlating with the number of vertebral fractures (18). In our study, OVCF patients treated with PVP/PKP had a 2-year postoperative NVCF probability of 15.74% (37/235), within the reported range of 2–52%. According to the Nomogram results, spinal CT value is the most significant factor influencing NVCF occurrence. Interestingly, as the number of cases increased, paraspinal muscle SMI emerged as a protective factor against NVCF, with Delong test confirming its non-significant impact. This contrasts with previous findings on the significant influence of paraspinal muscle parameters in NVCF occurrence (19–21). We assert that paraspinal muscle SMI, particularly multifidus SMI, holds predictive potential for NVCF. Confusion matrix, DCA, and CIC results suggest the model performs better in the low-incidence patient group, supported by pAUC results (pAUC_SP_ > pAUC_SE_).

Previous studies commonly employed BMD to assess osteoporosis severity, with DXA (2D planar projection technique) being the most widely used clinical method (22). However, DXA has limitations such as low resolution and an inability to directly image bone microstructure (e.g., cortical bone and trabeculae) (23). Its accuracy can be compromised, with error rates reaching up to 20% in patients with spinal deformities, degenerative diseases, or calcification, due to alterations in spinal structure (24). Thus, there is a need to explore novel methods for measuring BMD. In recent years, quantitative CT has garnered widespread attention for measuring spinal BMD. The Hounsfield Unit (HU) in CT provides a method for assessing local BMD, with studies demonstrating its correlation with DXA results (25). Research indicates that HU values measured by CT exhibit higher sensitivity and specificity (26). Additionally, according to Yang et al. (27) research, a low T-score may not necessarily be an independent risk factor for NVCF. This could be attributed to the prevalence of severe degenerative changes and compensatory osteophyte formation in the vertebrae of elderly patients with OVCF, potentially affecting the accuracy of T-scores. In contrast, spinal CT values, with their higher resolution, offer a better reflection of overall vertebral bone quality. Consequently, our study employed spinal CT values for BMD assessment and identified vertebral CT values as an independent protective factor for postoperative NVCF, aligning with the findings of Bian et al. (28).

As mentioned in the introduction, the spine, as a functional unit, exhibits close interactions between its skeletal structure and the paraspinal muscle system. The maximum mechanical load borne by the skeleton primarily results from the dynamic contraction activities of muscles. This dynamical stimulation can positively promote bone growth and remodeling by increasing periosteal tension caused by muscle mass and the traction effect of collagen fibers, thereby regulating bone density, enhancing bone strength and affecting bone microstructure (9, 29). Therefore, the decline of paraspinal muscle function will increase the risk of osteoporosis. Jeon et al. (30) revealed paraspinal muscle fat infiltration as a risk factor for vertebral collapse in OVCF patients. Additionally, Cheng et al. (21) found that post-PKP paraspinal muscle SMI at the L4 level is an independent protective factor against NVCF (OR 0.830). To account for varying patient heights, we standardized muscle FCSA across different individuals, as muscle volume typically correlates with patient height. Given the prevalent degenerative changes in vertebrae among the elderly, resulting in diffuse hypertrophy and an increase in vertebral CSA, this could potentially compromise the accuracy of assessing the muscle-vertebra index. Therefore, we employed SMI as a parameter for assessing muscle mass. Considering the functional variations among different paraspinal muscles, we conducted an analysis of the impact of each paraspinal muscle SMIs on NVCF. Our analysis revealed that multifidus SMI is an independent protective factor against NVCF after PVP/PKP. Consistent with Lee et al. (31), who found no difference in average CSA of multifidus between OVCF with osteoporosis, OVCF with bone loss, and the non-OVCF groups. However, multifidus FCSA in the OVCF group was significantly lower than the non-OVCF group. Logistic regression analysis indicated that multifidus FCSA at L4-5 and L5-S1 levels serves as a protective factor influencing OVCF. Additionally, as Chen et al. (12) discovered, asymmetry in multifidus CSA and fat infiltration rate are independent risk factors for adjacent segment NVCF after PVP. In the correlation analysis, we observed a significant negative correlation between multifidus SMI in NVCF patients and the fracture compression percentage (R = −0.331; *p* = 0.045). The potential association between decreased multifidus SMI and the occurrence of NVCF can be elucidated from two perspectives: (1) Sagittal plane imbalance. Sagittal balance enables the body to maintain overall center of gravity stability with minimal energy consumption, relying on the coordinated interaction of pelvic, lumbar vertebrae, and other skeletal morphologies. Lumbar lordosis (LL) plays a crucial role in maintaining sagittal plane balance (32). Multifidus, a deep spinal muscle and a major trunk extensor, exhibits the largest CSA in the lumbar region, primarily responsible for stabilizing the spine and maintaining lumbar curvature (33). Its contraction increases LL, and the significant negative correlation between multifidus SMI and LL, caused by multifidus fat infiltration, hampers sagittal plane balance maintenance, particularly in OVCF patients (34, 35). Decompensated sagittal balance may lead to unstable gait, increased fall risk, and thereby trigger NVCF. (2) Impeded fracture healing progression. According to Takahashi et al. (36) 6-month follow-up study on OVCF patients, a decrease in paraspinal muscle (multifidus and erector spinae) SFCSA is closely associated with delayed fracture healing. Poor fracture healing not only prolongs the postoperative recovery period for OVCF but also exacerbates the risk of subsequent NVCF. The strength gradient formed between the cement-fixed vertebra and its adjacent vertebra intensifies the occurrence of NVCF if delayed healing and insufficient contraction strength of paraspinal muscles fail to effectively compensate for the local stress distribution changes (21). In our correlation analysis between paraspinal muscle SMI and other preoperative factors, we observed a positive correlation with hemoglobin. Despite the low correlation coefficients (R = 0.150–0.466), this trend was evident in both all patients and those with NVCF. Future research could delve deeper into the relationship between them.

In this study, the surgical approach emerged as one of the predictive factors for NVCF. Both PKP and PVP are minimally invasive procedures widely utilized in treating OVCF, proven to be safe and effective. Their fundamental mechanism involves injecting bone cement into the fractured vertebra, restoring mechanical stability, enhancing strength, and alleviating pain symptoms (37). However, the assessment of postoperative efficacy between PVP and PKP remains contentious in clinical practice. Griffoni et al. (38) conducted a long-term follow-up study comparing the effectiveness and safety of PKP and PVP in treating OVCF. The results that while both procedures effectively restored vertebral height and improved spinal kyphosis, the risk of adjacent-segment NVCF was significantly higher in the PVP group than in the PKP group. Furthermore, Zhu et al. (39) demonstrated, in contrast to PVP and conservative treatment, PKP yielded superior improvements in quality of life and reduced the risk of postoperative NVCF in OVCF patients. Thus, PKP is considered the ideal choice for OVCF treatment, with a significantly lower incidence of adjacent vertebral fractures post-PKP. Consistent with our study’s findings, the Nomogram results demonstrate a reduced probability of NVCF occurrence in patients undergoing PKP treatment.

This study has several limitations. Firstly, this single-center retrospective study demonstrated good consistency within and between groups, validated externally. When the training set was split 8:2, Hosmer-Lemeshow goodness-of-fit tests for the training, testing, and validation sets all indicated acceptable model fits (all *p* > 0.05). However, as the training set proportion increased, a decreasing trend in the *p*-values of the Hosmer-Lemeshow test was observed. Thus, prospective large-scale clinical cohort studies are still needed for validation. Secondly, only three predictive variables were included, excluding many other factors such as physical activity. Future research should address these limitations and explore the impact of additional relevant factors.

## Data availability statement

The raw data supporting the conclusions of this article will be made available by the authors, without undue reservation.

## Ethics statement

The studies involving humans were approved by the Medical Ethics Committee of Hefei Hospital Affiliated to Anhui Medical University. The studies were conducted in accordance with the local legislation and institutional requirements. The ethics committee/institutional review board waived the requirement of written informed consent for participation from the participants or the participants’ legal guardians/next of kin because this study is retrospective. Ethical approval for the study was obtained from the Medical Ethics Committee of Hefei Hospital Affiliated to Anhui Medical University, and the need for informed consent was waived by the Medical Ethics Committee of Hefei Hospital Affiliated to Anhui Medical University.

## Author contributions

MT: Data curation, Formal analysis, Investigation, Methodology, Resources, Writing – original draft, Writing – review & editing. GZ: Data curation, Formal analysis, Investigation, Methodology, Resources, Writing – original draft, Writing – review & editing. FZ: Investigation, Writing – original draft, Writing – review & editing. XC: Investigation, Writing – original draft, Writing – review & editing. QF: Investigation, Writing – original draft, Writing – review & editing. MH: Investigation, Writing – original draft, Writing – review & editing. SY: Funding acquisition, Resources, Supervision, Validation, Writing – original draft, Writing – review & editing.
