# Supplementary material for: Paraspinal muscle parameters’ predictive value for new vertebral compression fractures post-vertebral augmentation: Nomogram development and validation
Source: Front Med (Lausanne). 2024 May 15;11:1379078. doi: 10.3389/fmed.2024.1379078 (PMC11133621; doi:10.3389/fmed.2024.1379078)

Supplementary Material

# Supplementary Data

**Supplementary** **information**

1. MRI Parameters

2. Surgical Approach

3. Statistical Analysis Details

4. Process of dividing the Training and Testing sets

**Supplementary tables**

1. Table S1 Characteristics of NVCF and No- NVCF Groups

2. Table S2 The intra- and inter-observer Correlation Coefficient of The Muscle Parameters of The Two Readers

3. Table S3 Correlation of Paraspinal Muscle SMI with Preoperative Factors

4. Table S4 Training and testing sets division

5. Table S5 Nomogram Predicts the Diagnostic Performance of NVCF Based on Subgroups

**Supplementary figures**

1. Figure S1 Radiographic Features and Spinal CT Values of NVCF

2. Figure S2 Comparison of partial AUC between the 6:4 and 8:2 models

3. Figure S3 Comparison of ROC of Nomogram and Other Independent Factors

4. Figure S4 Transparent Reporting of a Multivariable Prediction Model for Individual Prognosis or Diagnosis (TRIPOD)

# Supplementary information

**S1. MRI Parameters**

All patients underwent 3.0T MRI examination (Signa HDxt, Siemens, Germany) before surgery, and T2-weighted axial images were obtained. The relevant parameters were TR/TE=3200/950, slice thickness 4mm, field of view 260mm, matrix 256±512.

**S2. Surgical Approach**

Patients assumed a prone position for vertebral injury localization using fluoroscopy. Markings were made 3-4 cm to the left of the midline. Standard disinfection was followed by local infiltration anesthesia (5% lidocaine for regional nerve block). Under fluoroscopy, the puncture needle was positioned above and outside the projected pedicle, puncturing along the pedicle direction.

Percutaneous vertebroplasty (PVP) Group: The needle was slowly advanced to two-thirds anterior position, a working cannula was inserted, and bone cement was prepared and injected, ensuring even dispersion within the vertebra. After cement injection, the needle and cannula were removed, and cement leakage was monitored.

Percutaneous kyphoplasty (PKP) Group: After puncture, a bone drill was used to penetrate 3 mm into the anterior wall. A high-pressure balloon was inserted, inflated with contrast agent, and expanded to raise the collapsed vertebral margins. After restoring the vertebral height, cement was injected and cured before withdrawing the cannula under fluoroscopy.

**S3. Statistical Analysis Details**

Statistical analyses were conducted using R Studio software (v4.2.3, http://www.rproject.org/). Shapiro-Wilk test assessed normality for continuous data. Normally distributed data were presented as mean ± standard deviation (*±s*) and analyzed using independent sample t-tests. Non-normally distributed data were presented as median (*P25, P75*) and analyzed using the Mann-Whitney *U* test. Count data were presented as frequencies (%) and analyzed using *χ^2^* tests or Fisher's exact tests, followed by Bonferroni correction. Intra- and inter-observer correlation coefficients were computed.

Variable selection for new vertebral compression fracture (NVCF) occurrence utilized the Least Absolute Shrinkage and Selection Operator (LASSO) and Logistic Regression. Receiver Operating Characteristic (ROC) curves, Area Under the Curve (AUC), and Partial ROC (pROC) were plotted using the "pROC" package to assess model accuracy. Delong test and Bootstrap resampling (5000 iterations) were used to obtain *P*-values.

Calibration curves, goodness-of-fit tests (Hosmer-Lemeshow), and Decision Curve Analysis (DCA) were generated using the "rms" package to evaluate calibration and clinical utility. Results were presented through a Nomogram using the "nomogramFormula" package, uploaded to a webpage via the "rsconnect" package. Statistical significance was set at *P* < 0.05.

**S4. Process of dividing the Training and Testing sets**

In this study, we utilized data from 235 patients, employing four different data split ratios (5:5, 6:4, 7:3, and 8:2) to create training and testing sets. Lasso regression and logistic regression models were applied to different training set ratios for model development. Comparative analysis revealed similar AUC values for the 6:4 and 8:2 ratios in the test set (Delong test, *P* = 0.215). The 8:2 model incorporated muscle parameters (Supplementary Table S3).

To further explore the performance differences between these two models, we employed partial Area Under the Curve (pAUC) comparison. At a specificity threshold of 1-0.75, the 6:4 model's pAUC was 0.133 (95% *CI* 0.084, 0.179), and the 8:2 model's pAUC was 0.142 (95% *CI* 0.102, 0.184), with a Bootstrap *P*-value of 0.783 after 5000 iterations, indicating no significant difference within this specificity range. Similarly, with an changed specificity threshold to 1-0.8, the 6:4 model's pAUC was 0.095 (95% *CI* 0.054, 0.136), and the 8:2 model's pAUC was 0.106 (95% *CI* 0.074, 0.140), with a Bootstrap *P*-value of 0.695 (Supplementary Figure S3), demonstrating no statistically significant difference between the two.

For sensitivity assessment, within the 1-0.75 range, the 6:4 model's pAUC was 0.110 (95% *CI* 0.070, 0.187), and the 8:2 model's pAUC was 0.097 (95% *CI* 0.048, 0.169), with a Bootstrap *P*-value of 0.771. When changing the sensitivity threshold to 1-0.8, the 6:4 model's pAUC was 0.078 (95% *CI* 0.050, 0.145), and the 8:2 model's pAUC was 0.062 (95% *CI* 0.034, 0.126), with a Bootstrap *P*-value of 0.644 (Supplementary Figure S3). This indicates that at high specificity, the 8:2 model outperforms the 6:4 model in pAUC, while at high sensitivity, the 6:4 model outperforms the 8:2 model, although neither difference reaches statistical significance.

# Supplementary tables

**Table S1. Characteristics of NVCF and No- NVCF Groups**

| Characteristic | **No-NOVCF**  (*n*=198) | **NOVCF**  (*n*=37) | *P* |
| --- | --- | --- | --- |
| **Age (year)** | 74.67±7.99 | 77.03±7.62 | 0.098 |
| **Gender (male)** | 48(25.17%) | 5(19.57%) | 0.223 |
| **Body weight (kg)** | 56.77±9.89 | 54.43±10.64 | 0.194 |
| **BMI(kg/m²)** | 22.24±3.39 | 21.48±4.37 | 0.231 |
| **Diabetes (yes)** | 31(14.71%) | 6(19.12%) | 1.000 |
| **Hypertension (yes)** | 96(48.30%) | 16(39.13%) | 0.684 |
| **Smoking (yes)** | 11(5.10%) | 1(2.17%) | 0.751 |
| **Drinking (yes)** | 23(11.90%) | 1(4.35%) | 0.178 |
| **Pre-VAS score*** | 3(3, 5) | 4(3, 5) | 0.935 |
| **Post-VAS score*** | 2(1, 3) | 2(2, 3) | 0.831 |
| **Educational level** |  |  | 0.477 |
| Primary school≤ | 155(76.87%) | 30(73.91%) |  |
| Secondary school | 1(0.68%) | 1(2.17%) |  |
| High school | 30(17.35%) | 5(17.39%) |  |
| ≥College | 12(5.10%) | 1(6.52%) |  |
| **Occupation** |  |  | 0.723 |
| Farmer | 74(37.07%) | 11(28.26%) |  |
| Laborer | 5(2.72%) | 1(2.17%) |  |
| Self-employed households | 5(2.38%) | 0(2.17%) |  |
| Retirement | 81(41.84%) | 17(47.83%) |  |
| Other | 33(15.99%) | 8(19.57%) |  |
| **Time to first ambulation (day)** | 2(1, 3) | 2(1, 3) | 0.536 |
| **Osteoporosis medication** | 33(14.63%) | 7(17.39%) | 0.923 |
| **Surgical approach** |  |  | 0.065 |
| PVP | 67(32.65%) | 19(54.35%) |  |
| PKP | 131(67.35%) | 18(45.65%) |  |
| **Puncture** **pathway** |  |  | 0.388 |
| single | 38(18.71%) | 10(30.43%) |  |
| both | 160(81.29%) | 27(69.57%) |  |
| **Volume of injected bone cement (ml) *** | 5(3.63, 5) | 5(4, 5) | 0.838 |
| **Duration of surgery (min)** | 42.55±15.19 | 50.62±18.56 | **0.012**^#^ |
| **Multiple vertebral fractures** | 31(14.63%) | 8(19.57%) | 0.513 |
| **Fracture segment** |  |  | 0.566 |
| T10≤ | 29(9.86%) | 3(6.52%) |  |
| T11-L2 | 145(74.83%) | 29(69.57%) |  |
| L3-L5 | 24(15.31%) | 5(23.91%) |  |
| **Fracture compression (%)** | 40.29±11.42 | 43.19±12.33 | 0.163 |
| **Fracture shape** |  |  | **0.034**^#^ |
| Wedge | 119(54.76%) | 24(56.52%) |  |
| Biconcave | 78(44.56%) | 11(36.96%) |  |
| Crush | 1(0.68%) | 2(6.52%) |  |
| **IVC** | 46(22.11%) | 14(36.96%) | 0.096 |
| **Spinal CT values (HU)** | 62.46±30.22 | 30.80(14.51, 58.57) | **<0.001**^#^ |
| **AVHRR(%)*** | 10.70(4.20, 18.29) | 12.01(5.18, 20.17) | 0.637 |
| **Cobb angle change (°)*** | 2.24(0.51, 4.34) | 1.77(0.86, 3.14) | 0.554 |
| **Cement leakage (yes)** | 81(40.14%) | 16(41.30%) | 0.934 |
| **Leukocyte (10^9^/L)** | 6.14±1.73 | 6.73±2.75 | 0.313 |
| **Hemoglobin (g/L)** | 120.60±14.99 | 121.20±14.86 | 0.953 |
| **Urea (mmol/L)*** | 6.27(5.11, 7.55) | 5.42(4.36, 7.18) | 0.064 |
| **Creatinine (μmol/L)** | 62.45±20.21 | 60.43±16.86 | 0.617 |
| **CAR*** | 0.15(0.07, 0.41) | 0.34(0.09, 1.28) | **0.017**^#^ |
| **SMI(mm²/m²)** |  |  |  |
| Multifidus | 146.06±58.16 | 132.53±71.41 | 0.212 |
| Erector spinae | 330.05±119.96 | 305.36±132.73 | 0.260 |
| Psoas major | 291.50±90.93 | 285.49±97.52 | 0.714 |

**BMI:** Body Mass Index; **VAS:** Visual analog scale; **PVP:** Percutaneous vertebroplasty; **PKP:** Percutaneous kyphoplasty; **IVC:** Intravertebral cleft; **AVHRR:** Anterior vertebral height recovery ratio; **CAR:** C-reactive protein/ albumin ratio; **SMI:** Skeletal muscle index.

^*^Median (*P25*, *P75*); ^#^statistical significance.

**Table S2. The intra- and inter-observer Correlation Coefficient of The Muscle Parameters of The Two Readers**

|  | Reader 1 | Reader 2 | Reader 2* | Intra-observer | Inter-observer |
| --- | --- | --- | --- | --- | --- |
| **Left CSA** |  |  |  |  |  |
| Multifidus | 718.13±212.05 | 717.08±206.91 | 736.12±208.80 | 0.917 | 0.884 |
| Erector spinae | 1215.97±218.02 | 1208.46±219.08 | 1186.66±214.14 | 0.921 | 0.922 |
| Psoas major | 843.58±232.28 | 848.13±212.55 | 847.36±251.54 | 0.929 | 0.878 |
| Multifidus fat | 388.27±203.44 | 386.99±208.06 | 382.87±218.81 | 0.919 | 0.874 |
| Erector spinae fat | 427.73±224.39 | 446.40±223.84 | 443.43±213.46 | 0.945 | 0.926 |
| Psoas major fat | 115.58±89.59 | 117.20±88.92 | 115.03±92.16 | 0.992 | 0.989 |
| **Right CSA** |  |  |  |  |  |
| Multifidus | 737.35±191.56 | 707.10±186.08 | 736.76±208.15 | 0.936 | 0.852 |
| Erector spinae | 1227.49±218.04 | 1216.97±210.86 | 1214.04±224.70 | 0.926 | 0.897 |
| Psoas major | 827.51±207.97 | 827.19±212.32 | 824.11±246.28 | 0.949 | 0.914 |
| Multifidus fat | 407.68±252.73 | 420.14±217.38 | 426.87±218.89 | 0.948 | 0.893 |
| Erector spinae fat | 463.19±242.74 | 442.96±239.76 | 426.08±271.08 | 0.944 | 0.912 |
| Psoas major fat | 121.99±79.43 | 121.98±81.17 | 120.40±82.64 | 0.992 | 0.977 |
| **SMI(mm^2^/m^2^)** |  |  |  |  |  |
| Multifidus | 138.91±57.56 | 136.19±60.56 | 140.83±66.04 | 0.923 | 0.870 |
| Erector spinae | 325.79±117.46 | 322.478±98.37 | 316.35±101.06 | 0.957 | 0.829 |
| Psoas major | 290.05±71.76 | 289.24±75.61 | 285.68±78.09 | 0.949 | 0.917 |

**CSA:** Cross-Sectional Area; **SMI:** Skeletal Muscle Index.

^*^ Second measurement results.

**Table S3. Correlation of Paraspinal Muscle SMI with Preoperative Factors**

|  | Multifidus SMI | Erector spinae SMI | Psoas major SMI |
| --- | --- | --- | --- |
|  |  |  |  |
| **All patients(n=235)** |  |  |  |
| Age | *R*=0.013  *P*=0.848 | *R*=0.023  *P*=0.728 | *R*=0.033  *P*=0.613 |
| BMI | *R*<0.001  *P*=0.992 | *R*=0.026  *P*=0.688 | *R*<0.001  *P*=0.251 |
| CAR | *R*=0.042  *P*=0.525 | *R*=0.004  *P*=0.951 | *R*=0.071  *P*=0.279 |
| Fracture compression | *R*<0.001  *P*=0.992 | *R*=0.028  *P*=0.667 | *R*<0.001  *P*=0.822 |
| Creatinine | *R*=0.101  *P*=0.123 | ***R*=0.162**  ***P*=0.013** | ***R*=0.247**  ***P*<0.001** |
| Spinal CT values | *R*=0.076  *P*=0.246 | *R*<0.001  *P*=0.677 | *R*=0.082  *P*=0.210 |
| Hemoglobin | ***R*=0.150**  ***P*=0.021** | ***R*=0.212**  ***P*=0.001** | ***R*=0.269**  ***P*<0.001** |
| Urea | *R*=0.082  *P*=0.212 | ***R*=0.132**  ***P*=0.044** | *R*=0.046  *P*=0.479 |
| Leukocyte | *R*<0.001  *P*=0.401 | *R*=0.075  *P*=0.254 | *R*=0.038  *P*=0.566 |
| Body weight | *R*=0.016  *P*=0.812 | *R*<0.001  *P*=0.529 | *R*<0.001  *P*=0.321 |
| **NVCF patients(n=37)** |  |  |  |
| Age | *R*=0.132  *P*=0.437 | *R*=0.039  *P*=0.818 | *R*<0.001  *P*=0.944 |
| BMI | *R*=0.166  *P*=0.326 | *R*=0.146  *P*=0.388 | *R*=0.031  *P*=0.856 |
| CAR | *R*=0.174  *P*=0.302 | *R*=0.063  *P*=0.712 | *R*=0.117  *P*=0.488 |
| Fracture compression | ***R*=-0.331**  ***P*=0.045** | *R*<0.001  *P*=0.379 | *R*<0.001  *P*=0.380 |
| Creatinine | *R*=0.233  *P*=0.166 | *R*=0.243  *P*=0.147 | ***R*=0.362**  ***P*=0.028** |
| Spinal CT values | *R*=0.277  *P*=0.097 | *R*=0.049  *P*=0.775 | ***R*=0.336**  ***P*=0.042** |
| Hemoglobin | *R*=0.320  *P*=0.054 | ***R*=0.466**  ***P*=0.004** | *R*=0.318  *P*=0.055 |
| Urea | *R*=0.109  *P*=0.520 | *R*=0.266  *P*=0.112 | *R*=0.172  *P*=0.308 |
| Leukocyte | *R*=0.230  *P*=0.171 | *R*=0.320  *P*=0.054 | *R*=0.238  *P*=0.156 |
| Body weight | *R*=0.224  *P*=0.183 | *R*=0.106  *P*=0.532 | *R*=0.037  *P*=0.826 |

**BMI:** Body Mass Index; **CAR:** C-reactive protein/ albumin ratio

**Table S4. Training and testing sets division**

|  | Model | AUC  (Training Set) | AUC  (Testing Set) | *P*  (Delong) |
| --- | --- | --- | --- | --- |
|  |  |  |  |  |
| **5:5** | Surgical approach+  Spinal CT values | 0.774  (0.655, 0.894) | 0.732  (0.592, 0.872) | 0.657 |
| **6:4** | Surgical approach+  Spinal CT values | 0.805  (0.697, 0.912) | 0.678  (0.514, 0.842) | 0.205 |
| **7:3** | Surgical approach+  Spinal CT values | 0.794  (0.690, 0.898) | 0.657  (0.464, 0.850) | 0.225 |
| **8:2** | Surgical approach+  Spinal CT values+  Multifidus muscle SMI | 0.801  (0.698, 0.905) | 0.664  (0.475, 0.853) | 0.215 |

**Table S5. Nomogram Predicts the Diagnostic Performance of NVCF Based on Subgroups**

|  | AUC  (95%CI) | Accuracy | Sensitivity  (95%CI) | Specificity  (95%CI) | PPV  (95%CI) | NPV  (95%CI) |
| --- | --- | --- | --- | --- | --- | --- |
| **Sex** |  |  |  |  |  |  |
| Male | 0.738  (0.449, 1.000) | 0.736 | 0.800  (0.284, 0.995) | 0.729  (0.582, 0.847) | 0.235  (0.068, 0.499) | 0.972  (0.855, 0.999) |
| Female | 0.755  (0.650, 0.860) | 0.687 | 0.719  (0.533, 0.863) | 0.680  (0.599, 0.754) | 0.324  (0.218, 0.445) | 0.919  (0.852, 0.962) |
| **BMI(kg/m²)** |  |  |  |  |  |  |
| ≤24 | 0.790  (0.675, 0.905) | 0.854 | 0.630  (0.424, 0.806) | 0.898  (0.834, 0.943) | 0.548  (0.360, 0.727) | 0.925  (0.866, 0.963) |
| ＞24 | 0.703  (0.547, 0.860) | 0.634 | 0.800  (0.444, 0.975) | 0.607  (0.473, 0.729) | 0.250  (0.115, 0.434) | 0.949  (0.827, 0.994) |
| **Duration of surgery**  **(min)** |  |  |  |  |  |  |
| ≤40 | 0.775  (0.628, 0.923) | 0.795 | 0.706  (0.440, 0.897) | 0.809  (0.725, 0.876) | 0.353  (0.197, 0.535) | 0.949  (0.885, 0.983) |
| ＞40 | 0.758  (0.629, 0.888) | 0.748 | 0.750  (0.509, 0.913) | 0.747  (0.640, 0.836) | 0.417  (0.255, 0.592) | 0.925  (0.834, 0.975) |
| **Cement leakage** |  |  |  |  |  |  |
| Yes | 0.836  (0.737, 0.934) | 0.814 | 0.750  (0.476, 0.927) | 0.827  (0.727, 0.902) | 0.462  (0.266, 0.666) | 0.944  (0.862, 0.984) |
| No | 0.745  (0.601, 0.889) | 0.790 | 0.714  (0.478, 0.887) | 0.803  (0.720, 0.871) | 0.395  (0.240, 0.566) | 0.940  (0.874, 0.978) |
| **Time to first ambulation**  **(day)** |  |  |  |  |  |  |
| ≤3 | 0.756  (0.650, 0.863) | 0.750 | 0.710  (0.520, 0.858) | 0.758  (0.685, 0.821) | 0.355  (0.237, 0.487) | 0.933  (0.876, 0.969) |
| ＞3 | 0.753  (0.510, 0.995) | 0.897 | 0.500  (0.118, 0.882) | 0.970  (0.842, 0.999) | 0.750  (0.194, 0.994) | 0.914  (0.769, 0.982) |
| **IVC** |  |  |  |  |  |  |
| Yes | 0.837  (0.684, 0.990) | 0.900 | 0.714  (0.419, 0.916) | 0.957  (0.852, 0.995) | 0.833  (0.516, 0.979) | 0.917  (0.800, 0.977) |
| No | 0.717  (0.593, 0.840) | 0.789 | 0.609  (0.385, 0.803) | 0.816  (0.745, 0.874) | 0.333  (0.196, 0.495) | 0.932  (0.875, 0.969) |

Accuracy, sensitivity, and specificity are all obtained by evaluating the performance using the cutoff value when the Youden index is maximized. The subgroup analysis was conducted based on gender, with a Delong test yielding a *P*-value of 0.911; for BMI (24 kg/m²), the *P*-value was 0.384; for duration of surgery (40 min), the *P*-value was 0.865; for cement leakage, the *P*-value was 0.310; for time to first ambulation (3 days), the *P*-value was 0.977; and for IVC, the *P*-value was 0.232. **AUC:** Area under the receiver operating characteristic curve; **PPV:** Positive Predictive Value; **NPV:** Negative Predictive Value.

# Supplementary figures


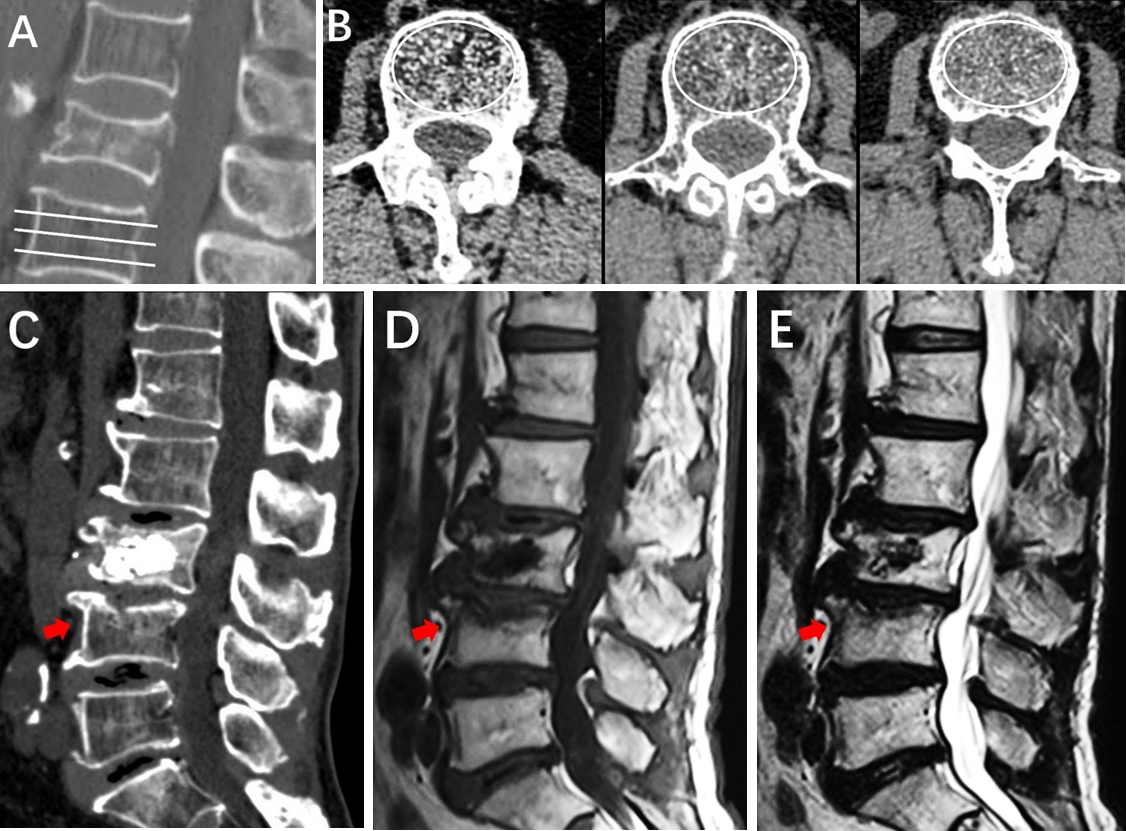
**Figure S1. Radiographic Features and Spinal CT Values of NVCF**

The Hounsfield unit of the vertebra in the segment adjacent to the fractured vertebra. **A)** Trisecting the vertebral body in the sagittal plane using three-dimensional CT reconstruction of the spine; **B)** Three-dimensional CT reconstruction of the transverse section of the spine; **C, D, E)** NVCF exhibits low signal intensity on T1-weighted images and high signal intensity on T2-weighted images.


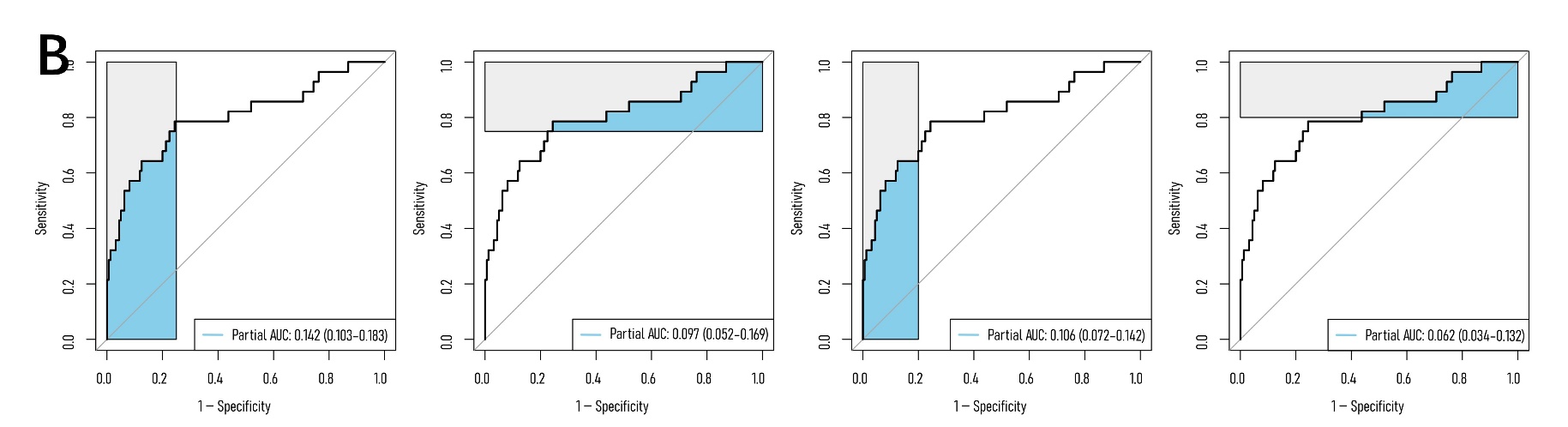

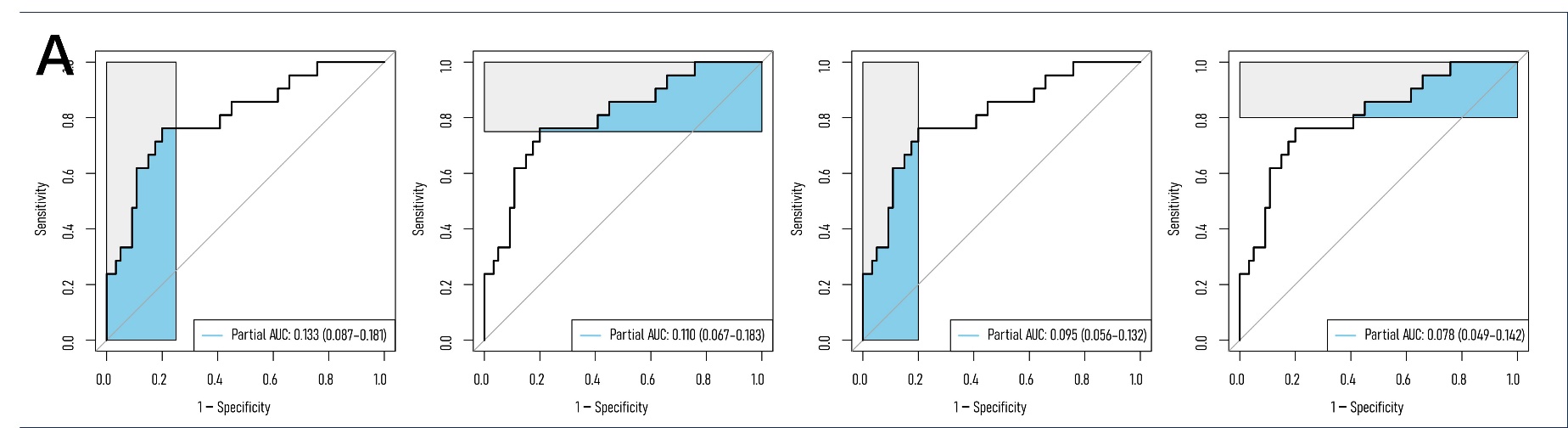
**Figure S2. Comparison of partial AUC between the 6:4 and 8:2 models**

**A)** pAUC of the 6:4 model at different specificities and sensitivities. Left two figures represent 1-0.75 specificity and sensitivity, and the right two figures represent 1-0.80 specificity and sensitivity. **B)** pAUC of the 8:2 model at different specificities and sensitivities. Left two figures represent 1-0.75 specificity and sensitivity, and the right two figures represent 1-0.80 specificity and sensitivity.


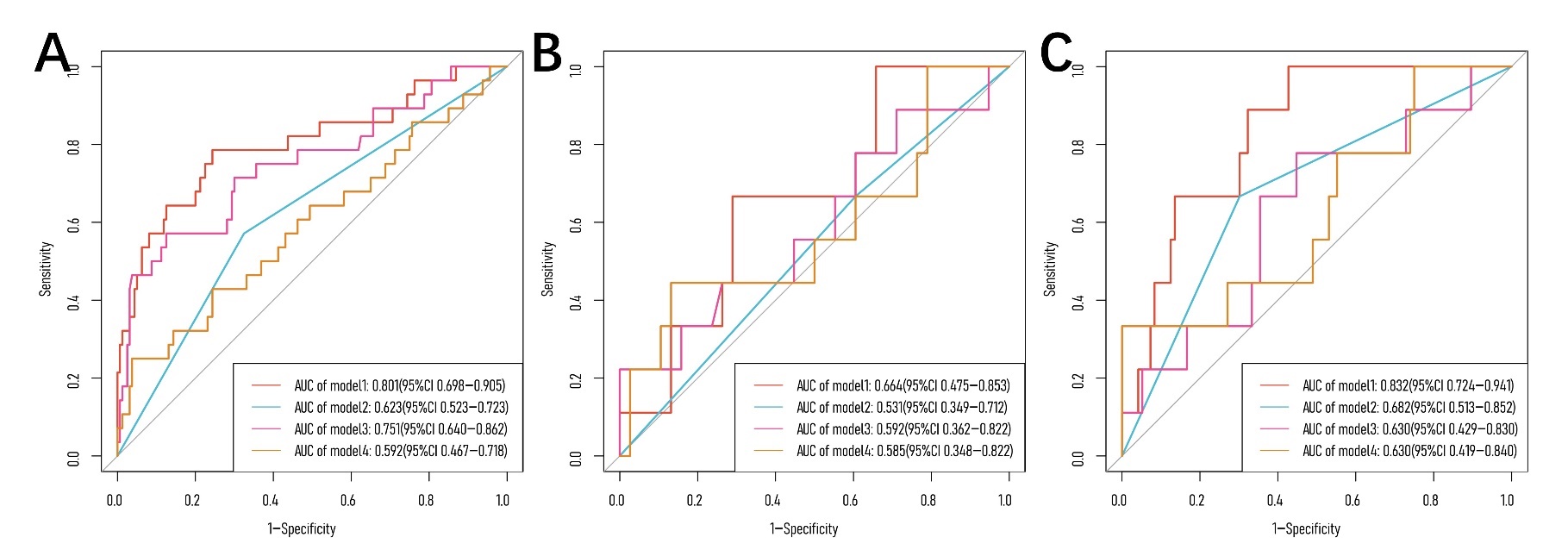
**Figure S3. Comparison of ROC of Nomogram and Other Independent Factors**

ROCs of nomogram and other independent factors (training set: A, testing set: B, validation set: C). Model1: Nomogram (Surgical approach + Spinal CT values + Multifidus SMI); Model2: Surgical approach; Model3: Spinal CT values; Model4: Multifidus SMI.

**Figure S4. Transparent Reporting of a Multivariable Prediction Model for Individual Prognosis or Diagnosis (TRIPOD)**


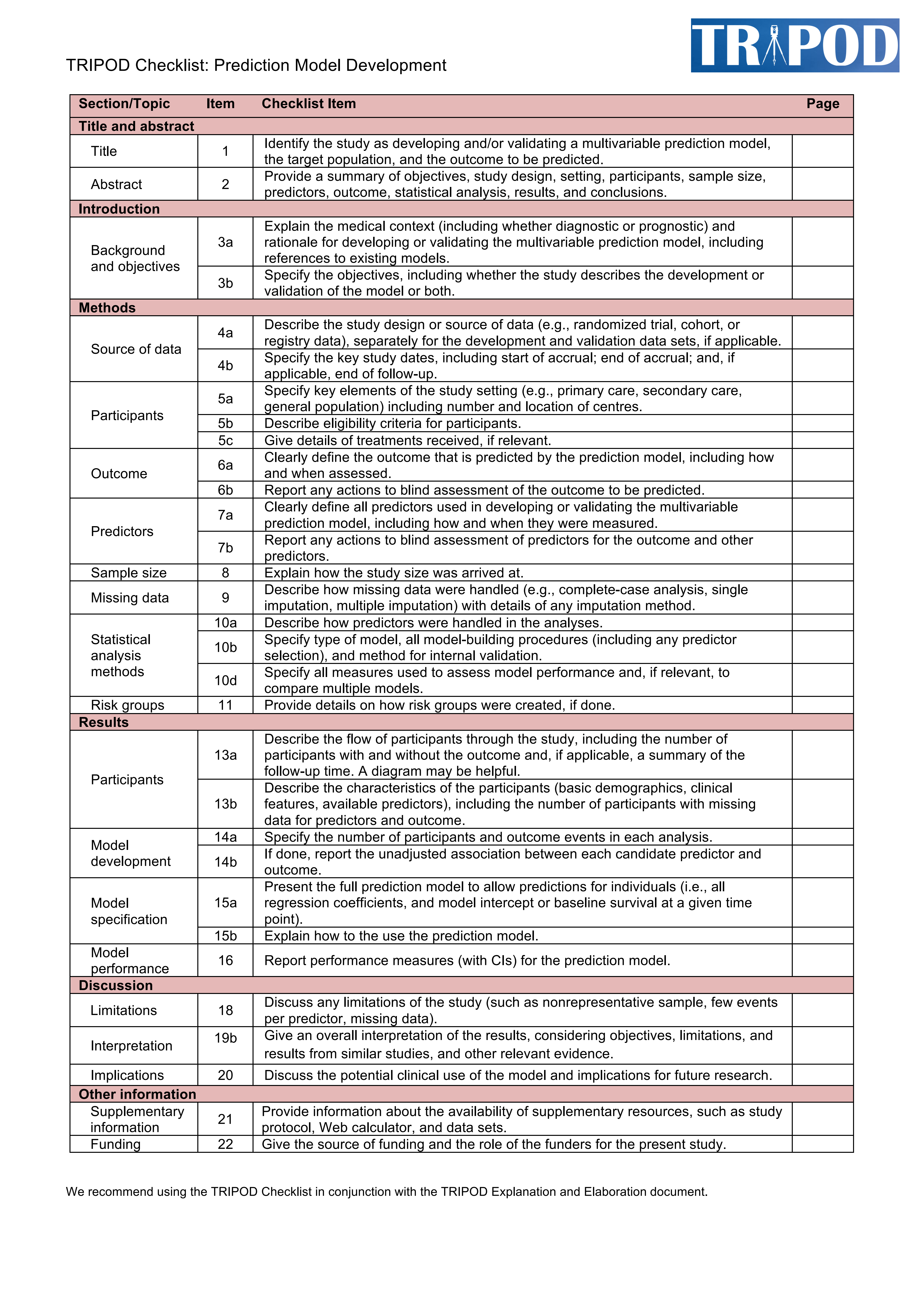

Supplement: Supplementary file 1 [file Data_Sheet_1.docx]
